# Congenital Factor VII Deficiency: A Case Study of Four Family Members

**DOI:** 10.7759/cureus.76595

**Published:** 2024-12-29

**Authors:** Smail Ghouzraf, Hicham Yahyaoui, Mohamed Chakour

**Affiliations:** 1 Hematology, Avicenna Military Hospital, Marrakesh, MAR

**Keywords:** autosomal recessive inheritance, factor vii deficiency, hemostasis disorders, rare coagulation factor deficiency, recombinant fvii

## Abstract

Congenital factor VII (FVII) deficiency is a rare genetic disorder with autosomal recessive inheritance, characterized by molecular and clinical heterogeneity. This article reports four Moroccan cases of FVII deficiency within the same family, two of which were associated with Gilbert's syndrome.

The index case was a 15-year-old girl with a history of menorrhagia and jaundice. Upon examination, she presented with indirect hyperbilirubinemia, iron deficiency anemia, and a prolonged prothrombin time (PT). FVII deficiency was confirmed with a factor VII level of 45%. Her mother, a 30-year-old woman with sarcoidosis and a history of postpartum hemorrhage, was found to have jaundice and an FVII deficiency at 42%. The brother of the index case, an eight-year-old boy who was asymptomatic, was discovered to have FVII deficiency during family screening, with a level of 41%. Similarly, the six-year-old sister, also asymptomatic, had an FVII level of 33%.

The prevalence of homozygous FVII deficiency is rare, and the risk is increased in consanguineous marriages. Clinical presentations vary widely, ranging from asymptomatic cases to severe bleeding episodes. Diagnosis is based on a prolonged PT with a normal activated partial thromboplastin time (aPTT) and is confirmed through FVII assays. Severe cases may require prophylactic treatment, and recombinant activated FVII (rFVIIa) is the recommended therapy for bleeding episodes.

In conclusion, FVII deficiency is the most common of the rare coagulation factor deficiencies. This study explores familial congenital factor VII deficiency, characterized by varied presentations from asymptomatic to mild hemorrhagic symptoms. Early diagnosis and family screening are essential. Management includes symptomatic treatment, tranexamic acid for menorrhagia, and fresh frozen plasma or recombinant factor VIIa for bleeding episodes.

## Introduction

Coagulation factor VII is a vitamin K-dependent glycoprotein synthesized in the liver. Congenital factor VII deficiency is the most common of the rare coagulation factor deficiencies (RCFDs), first described in 1951 by Dr. Alexander B. and colleagues. It is a genetic disorder with autosomal recessive inheritance and variable penetrance, caused by a mutation in the gene encoding FVII [1]. The disorder is characterized by significant molecular heterogeneity, which leads to a wide spectrum of mutations and highly variable clinical presentations [2]. We present four Moroccan cases of congenital FVII deficiency identified within the same family, two of whom also exhibited an association with Gilbert's syndrome.

## Case presentation

The index case of our study is a young patient aged 15 years, from a non-consanguineous marriage, with a history of two episodes of moderate skin and mucosal jaundice and menorrhagia (menarche at 13 years). Familially, her mother had sarcoidosis. The patient was admitted due to bilateral arthralgias of an inflammatory nature affecting the knee, hip, and elbow, persisting for 10 days prior to admission, associated with polypnea, night sweats, and a non-cholestatic skin and mucosal jaundice, in the context of undetermined fever and a general health deterioration characterized by asthenia and anorexia without weight loss.

On clinical examination, joint examination revealed pain upon palpation of the elbows, hips, and knees bilaterally. Lymphatic and abdominal pelvic examinations were normal. The remainder of the somatic examination was unremarkable.

Radiologically, an abdominal-pelvic ultrasound was performed, revealing a liver with a homogenous echotexture, normal morphology, and size, with no hepatic hilar or coelio-mesenteric lymphadenopathies. The gallbladder was free of calculi and had a thin wall. Doppler study demonstrated complete patency of the hepatic veins, which appeared slender with a multiphasic flow. The inferior vena cava was patent, without any morphological or hemodynamic abnormalities. The pelvic region showed no abnormalities. In conclusion, the abdominal ultrasound findings were within normal limits.

Additionally, a frontal chest X-ray was performed, revealing bilateral parenchymal opacities predominantly in the perihilar regions, along with peribronchovascular thickening. This presentation was suggestive of an infectious pneumonia, likely of viral origin.

Biologically, a complete liver function test revealed indirect hyperbilirubinemia. A complete blood count detected hypochromic microcytic anemia. Serum ferritin was decreased to 10 µg/L (normal range: 15-150 µg/L), allowing the diagnosis of iron deficiency anemia. Inflammatory, thyroid, and renal assessments were normal.

The diagnosis of Gilbert's syndrome was retained for this patient based on clinico-biological arguments: the first episode dating back to the age of 13 years, spontaneous resolution of jaundice in both episodes and normal growth parameters and puberty development. Biologically, the indirect hyperbilirubinemia was moderate, and the etiological workup aimed at excluding other causes of indirect bilirubin jaundice was normal.

Gilbert's syndrome was the circumstance that led to the discovery of factor VII deficiency in this patient. The hemostasis assessment showed a prothrombin time (PT) prolonged to 1.28 times the control time. The factor VII assay indicated a decreased level at 45% (control at 44%). The levels of coagulation factors II, V, and X were normal (Table 1). The search for anti-factor VII antibodies was negative. The congenital origin of the factor VII deficiency in this patient was confirmed after excluding any acquired causes (liver failure, vitamin K deficiency, hyperfibrinolysis, disseminated intravascular coagulation, and negative anti-factor VII antibodies).

**Table 1:** Biological assessment results of the index case showing a prolonged prothrombin time and decreased factor VII level at 45% PT: prothrombin time

Biological examination	Result	Reference values
Prothrombin time (sec)	17	Control: 13.3
PT (%)	61	70-100
Activated partial thromboplastin time (sec)	29.3	Control: 32
Fibrinogen (g/L)	2	2-4
Factor VII (%)	45	70-130
Factors II, V, X (%)	Normal	70-120
Hemoglobin (g/dL)	11.5	12-16
Platelets (µL)	187	150-450
Leucocytes (x 10³/mm³)	7.8	4-10

Regarding the menorrhagia experienced by this patient, a prescription was provided, including tranexamic acid at a dose of 20 mg/kg/day to be taken during her menstrual periods, along with an iron supplement to be taken for two months. The evolution was marked by an improvement in her menorrhagia; regular follow-up is still maintained, and the patient is in good general health.

The second case in our study was the mother of the index case, aged 34 years, from a non-consanguineous marriage, widowed, and mother of three children. She had been monitored for Löfgren's syndrome for seven years and had a history of a postpartum hemorrhage in 2015. The patient was called for a familial investigation in our study, where we observed non-cholestatic skin and mucosal jaundice evolving for a month and a half in the context of apyrexia and general health deterioration. Clinical examination revealed firm, painless, non-inflammatory cervical lymphadenopathy measuring 1.5 cm x 1 cm.

Biologically, the hemostasis assessment indicated a moderate prolongation of the PT (PT patient / PT control = 1.28), as well as factor VII deficiency with a decreased level at 42% (control at 44%). The levels of coagulation factors II, V, and X were normal. The liver function tests revealed indirect hyperbilirubinemia.

The third case involved the brother of the index case, who was eight years old and had no significant personal history. The patient was called for screening for a possible congenital factor VII deficiency. A hemostasis assessment was performed, revealing a minimal prolongation of the PT (PT patient / PT control at 1.21, decreased to 1.20 after control), along with normal activated partial thromboplastin time (aPTT) and fibrinogen levels. The factor VII assay indicated a decreased level at 41% (control at 42%). Given the presence of two similar cases in the family and the repeated decrease of factor VII in this patient, the congenital origin of the factor VII deficiency was confirmed. The remainder of the assessment, particularly liver function, was normal.

As part of the familial screening, we also summoned the sister of the index case, aged six years, with no significant personal history. A hemostasis assessment was conducted, revealing a moderate prolongation of the PT (PT patient / PT control at 1.25, decreased to 1.24 after control). The factor VII assay showed a decreased level at 33% (control at 32%).

The rest of the family members (uncle and grandmother of the index case) did not exhibit any abnormalities in factor VII levels.

## Discussion

The prevalence of homozygous congenital factor VII deficiency is estimated to be 1 in 500,000 in the global population, with a higher prevalence in countries where consanguineous marriage is common [3], which was not the case in our study. The average age of diagnosis in our four patients was 15 years. In other published studies, all age groups were represented, with a marked predominance of young adults [3-5]. In our index case, the deficiency was discovered during the investigation of a prolonged PT. The remaining three cases were identified through family screening. The index case had menorrhagia, unlike the rest of the family members. Similarly, in the study by Rami [3], the discovery was incidental, unlike in the study by Kada, where 67% of cases were revealed by hemorrhagic syndrome [6]. In the study conducted by Acharya, the modes of presentation were as follows: hemorrhagic manifestations in 46% of cases, investigation of prolonged PT in 40%, a positive family history in 12%, and thromboembolic manifestations in 2% [7].

Hemorrhagic syndrome associated with congenital factor VII deficiency is highly variable, ranging from cutaneous-mucosal bleeding in most patients to life-threatening severe hemorrhages in 10% to 15% of cases. Conversely, up to one-third of patients remain asymptomatic throughout their lives and are diagnosed during family investigations or preoperative hemostatic screening [8]. In a multicenter study conducted by Mariani et al., 36.7% of symptomatic patients presented with epistaxis, with higher rates reported by the studies of Herrmann et al. and Peyvandi et al. [9-11]. We did not observe any instances of epistaxis in our patients. The two symptomatic patients in our study presented with menorrhagia and postpartum hemorrhage, respectively. According to the international factor VII deficiency registry, 63% of women aged 10 to 50 years experienced menorrhagia, which aligns with our findings [12].

Factor VII deficiency can be associated with other coagulation factor deficiencies. Associations with metabolic disorders of bilirubin metabolism are rare but do exist, including Dubin-Johnson syndrome and Gilbert's disease, which were present in two of our patients.

A prolonged PT with a normal aPTT raises suspicion of factor VII deficiency. Confirmation is made through factor VII assays, with normal levels ranging from 70% to 140%. Several studies have confirmed this biological profile [3-5]. In our study, all PTs were prolonged, with an average of 16.37 seconds compared to a control of 13.3 seconds (international normalized ratio (INR) average: 1.3). The aPTT was normal.

Mariani et al. identified three levels of severity for factor VII deficiency, based on the presence or absence of hemorrhagic symptoms, classified as defining the severity of the deficiency (central nervous system bleeding and/or gastrointestinal bleeding and/or hemarthrosis) [9].

For factor VII assays in our patients, we used rabbit-derived thromboplastin (STA®-NeoPTimal⑩, Diagnostica Stago, France). The factor VII levels in our patients ranged between 33% and 44%. Fifty percent of our patients were asymptomatic, while 50% had a mild form of the disease (menorrhagia in one patient and a history of postpartum hemorrhage in another). None of the patients had experienced severe hemorrhagic episodes in their lifetime.

The lack of clinico-biological correlation makes it difficult to predict clinical severity based solely on factor VII levels. This can be attributed to technical variables, including the human or animal origin of the thromboplastin, the quality of the calibrator, or the type of reference plasma used [5]. However, the severity of initial hemorrhagic manifestations can serve as a predictor for the risk of future severe episodes [8].

Regarding genotype, a comparative study involving 84 heterozygous patients and a control group followed for 22.6 years showed symptomatic rates of 9.5% and 8.3%, respectively [13]. Similarly, in two cohorts reported in the literature, which included 128 and 499 heterozygous patients, the percentage of symptomatic individuals was 33% and 19%, respectively [9,10]. Severe hemorrhagic manifestations were more commonly observed in homozygous and compound heterozygous individuals, whereas mild clinical phenotypes were more frequent among heterozygotes [9].

On a genetic level, molecular studies by sequencing coding regions, exon/intron junctions, and the promoter of the F7 gene have identified over 250 different mutations, with 70-80% being missense mutations. Other mutations commonly affect splicing sites and include nonsense mutations or small deletions [14].

Genetic characterization of F7 mutations complements clinical evaluation and results from functional tests, particularly in uncertain cases. Molecular data must be interpreted alongside clinical symptoms and phenotypic analysis to avoid false-positive or false-negative results.

In terms of patient management, prophylaxis appears to be the most appropriate therapeutic option for patients with severe forms of the disease or in the event of surgical interventions. Regimens involving three weekly injections have been reported in the literature. In our series, none of the patients were eligible for prophylactic treatment due to the absence of severe forms. Long-term management of asymptomatic children with congenital factor VII deficiency requires individualized monitoring and preventive strategies. Regular hematologic evaluations to track FVII activity and coagulation parameters are essential. Medical follow-ups with hematologists should ensure readiness for procedures requiring hemostatic interventions, such as recombinant-activated FVII. Proactive psychosocial monitoring ensures the condition does not adversely affect the quality of life. This comprehensive approach enhances safety and promotes optimal outcomes for asymptomatic pediatric patients.

The prophylactic use of recombinant activated factor VII (rFVIIa - Novoseven®, Novo Nordisk, Denmark) in women with congenital factor VII deficiency during childbirth remains controversial. Most published studies suggest that women with factor VII levels below 10-20% are at high risk for postpartum hemorrhage and thus require prophylaxis, particularly if they have a history of hemorrhagic episodes [15].

In cases of hemorrhage related to factor VII deficiency, treatment involves substitution with fresh frozen plasma (FFP) or the administration of factor VII. Recombinant factor VIIa (rFVIIa) is highly effective and well-tolerated, with no reported infectious risks or thrombotic complications. The recommended dose is 15-30 µg/kg every 4-6 hours until hemorrhage is controlled. Due to the high cost of factor VII, we continue to administer FFP in symptomatic patients in cases of hemorrhage. FFP transfusion remains valuable in our context, despite its low efficacy and the risk of fluid overload.

In our index case, after ruling out any gynecological causes for the menorrhagia, treatment was based on the administration of tranexamic acid at a dose of 20 mg/kg/day, to be taken throughout the menstruation period.

## Conclusions

This study highlights a familial pattern of congenital factor VII deficiency, discovered in a young index case presenting with Gilbert's syndrome. The diagnosis was confirmed through prolonged PT and decreased factor VII levels, with family screening identifying additional cases. The clinical presentation ranged from asymptomatic to mild hemorrhagic manifestations, such as menorrhagia and postpartum hemorrhage. The condition's variable severity emphasizes the importance of individualized assessment, as clinical symptoms do not always correlate with factor VII levels. Management strategies focused on symptomatic treatment, with tranexamic acid effectively addressing menorrhagia in the index case. FFP remains a practical option for managing bleeding episodes in resource-limited settings, while rFVIIa offers a targeted approach in severe cases. This study underscores the importance of early diagnosis, family screening, and tailored management to optimize outcomes for patients with factor VII deficiency.
